# Exogenous 10 kDa-Heat Shock Protein Preserves Mitochondrial Function After Hypoxia/Reoxygenation

**DOI:** 10.3389/fphar.2020.00545

**Published:** 2020-05-05

**Authors:** Leonardo Maciel, Dahienne Ferreira de Oliveira, Gustavo Monnerat, Antonio Carlos Campos de Carvalho, Jose Hamilton Matheus Nascimento

**Affiliations:** ^1^Laboratory of Cardiac Electrophysiology Antônio Paes de Carvalho, Institute of Biophysics Carlos Chagas Filho, Federal University of Rio de Janeiro, Rio de Janeiro, Brazil; ^2^National Institute of Cardiology, Rio de Janeiro, Brazil

**Keywords:** ischemic preconditioning, mitochondria, HSP10, humoral factors, cardioprotection

## Abstract

Humoral factors released during ischemic preconditioning (IPC) protect the myocardium against ischemia/reperfusion (I/R) injury. We have recently identified 10 kDa-heat shock protein (HSP10) and a fraction of small 5–10 kDa peptides (5–10-sP) in the coronary effluent of IPC-treated hearts and demonstrated their cardioprotective potential. We here used our isolated mitochondria model to characterize the impact of exogenous HSP10 and 5–10-sP on mitochondria function from myocardium subjected to I/R injury. Isolated perfused rat hearts were submitted to 30-min global ischemia and 10-min reperfusion. Before ischemia, isolated hearts were infused with saline or 5–10-sP, with or without a mitochondrial ATP-sensitive-K^+^-channel blocker (5HD 10 μmol·L^−1^) or PKC inhibitor (chelerythrine 10 μmol·L^−1^), before I/R. HSP10 (1 µmol·L^−1^) was infused into isolated hearts before I/R without blockers. At 10-min reperfusion, the mitochondria were isolated and mitochondrial function was assessed. In a subset of experiments, freshly isolated mitochondria were directly incubated with HSP10 or 5–10-sP with or without 5HD or chelerythrine before *in vitro* hypoxia/reoxygenation. Infusion of 5–10-sP (n = 5) and HSP10 (n = 5) into isolated hearts before I/R improved mitochondrial ADP-stimulated respiration, ATP production and prevented mitochondrial ROS formation compared to the I/R group (n = 5); this effect was abrogated by 5HD and chelerythrine. In freshly isolated mitochondria with *in vitro* hypoxia/reoxygenation, HSP10 (n = 16) and 5–10-sP (n = 16) incubation prevented reductions of mitochondrial ADP-stimulated respiration (91.5 ± 5.1 nmol O2/min/mg PTN), ATP production (250.1 ± 9.3 μmol ATP/200μg PTN), and prevented mitochondrial ROS production (219.7 ± 9.0 nmol H2O2/200μg PTN) induced by hypoxia/reoxygenation (n = 12, 51.5 ± 5.0 nmol O2/min/mg PTN; 187 ± 21.7 μmol ATP/200 μg PTN; 339.0 ± 14.3 nmol H2O2/200 μg PTN, *p* < 0.001, respectively). 5HD reduced the ADP-stimulated respiration in the HSP10 group (65.84 ± 3.3 nmol O2/min/mg PTN), ATP production (193.7 ± 12.1 μmol ATP/200μg PTN) and increased ROS in the 5–10-sP group (274.4 ± 21.7 nmol H2O2/200 μg PTN). Mitochondria are a target of the cardioprotection induced by 5–10-sP and HSP10. This protection is dependent of PKC and mK_ATP_ activation. HSP10 can act directly on mitochondria and protects against hypoxia/reoxygenation injury by mK_ATP_ activation.

## Introduction

Ischemic preconditioning (IPC) is a cardioprotective maneuver constituted by brief episodes of ischemia/reperfusion (I/R) before sustained severe myocardial I/R (Murry et al., 1986; Hausenloy et al., 2016). IPC can be induced directly in the heart and also at distance in remote tissues and organs (remote ischemic preconditioning, RIPC) (Heusch et al., 2015a; Kleinbongard et al., 2017). Humoral and neuronal pathways are involved in IPC’s and RIPC’s cardioprotection (Basalay et al., 2018). IPC protection can be transferred humorally, among isolated hearts by coronary effluent transfer (Serejo et al., 2007) or *via* blood transfusion (Dickson et al., 1999a). Thus, a better understanding of how the IPC stimulus is transferred to the heart, as well as identification of its cellular targets in the protected organ, is necessary in order to ameliorate the use of IPC in patients (Dickson et al., 1999a; Dickson et al., 1999b; Heusch and Gersh, 2016; Heusch, 2017).

Mitochondria are listed as end-effectors of many cardioprotective maneuvers (Heusch and Gersh, 2017). Indeed, mitochondrial function preservation after I/R is decisive for the survival of cardiac cells and thus myocardium recovery (Heusch and Gersh, 2017). Changes in mitochondrial function by IPC or RIPC have been shown in several studies: mitochondrial respiration was improved in rat hearts (Heusch, 2015b; Kleinbongard et al., 2018) and in the right atrium of patients undergoing cardiac surgery (Heusch et al., 2015b). Recently, infarct size reduction, induced by RIPC, was associated with improved mitochondrial function at early reperfusion, supporting the concept that the mitochondria are an intracellular target of protection by RIPC (Heusch et al., 2010). However, despite the mitochondria being a target of the cardioprotective pathways activated by RIPC, signaling pathways for mitochondrial protection remain unclear (Gedik et al., 2017).

Recently we reported the presence of small, 5–10 kDa, peptides (5–10-sP) among the humoral factors released on coronary effluent from hearts submitted to IPC, as having cardioprotective effects (Maciel et al., 2017). Furthermore, we showed the activation of PKC and mitochondrial ATP-dependent potassium channel (K_ATP_) by 5–10-sP (Maciel et al., 2017). The activation of PKC is related to inhibit the mitochondrial permeability transition pore (MPTP) structuring and activate mitochondrial-K_ATP_ channels (K_ATP_), being this MPTP inhibition and K_ATP_ activation related to cell survival (Halestrap et al., 2007; Heusch, 2015b). Moreover, our proteomic data analysis revealed the presence of the 10 kDa-mitochondrial heat shock protein (HSP10) on 5–10-sP (Maciel et al., 2017). HSP10 is a cochaperonin, canonically involved in the import of mitochondrial proteins and in macromolecular assembly. Together with HSP60, it facilitates protein folding. It can also prevent incorrect folding and promote the proper assembly of nonfolded polypeptides, generated under stress conditions in the mitochondrial matrix (Viitanen et al., 1992; Cavanagh and Morton, 1994; Levy-Rimler et al., 2001). Regarding cell protection, HSP10 has already been shown to modulate intracellular cytoprotective signaling, activating the BCL-2 dependent pathway (Shan, 2003). In addition, protection by HSP10 has also been associated with folding or ubiquitination of proteins (Shan, 2003). Indeed, exogenous HSP10 perfusion reduced infarct size and improved the hemodynamic parameters in hearts submitted to I/R (Maciel et al., 2017).

Thus, the aims of the present study were (Murry et al., 1986) to evaluate whether 5–10-sP improve mitochondrial function and (Hausenloy et al., 2016) to evaluate whether HSP10 perfusion on isolated hearts and direct incubation of HSP10 with isolated mitochondria improve mitochondrial function.

## Methods

### Materials

All chemicals (analytical grade) were obtained from Sigma-Aldrich (USA) if not otherwise specified. All solutions were freshly prepared and filtrated (1.2 μm, Millipore). Wistar rats (male. 300–350 g, CCS-Central Animal Facility) were used following the Guide for the Care and Use of Laboratory Animals published by the US National Institutes of Health (8th edition, 2011) and the local Institutional Animal Care and Use Committee (100/16).

### Isolated Perfused Rat Hearts

**Figure 1:**
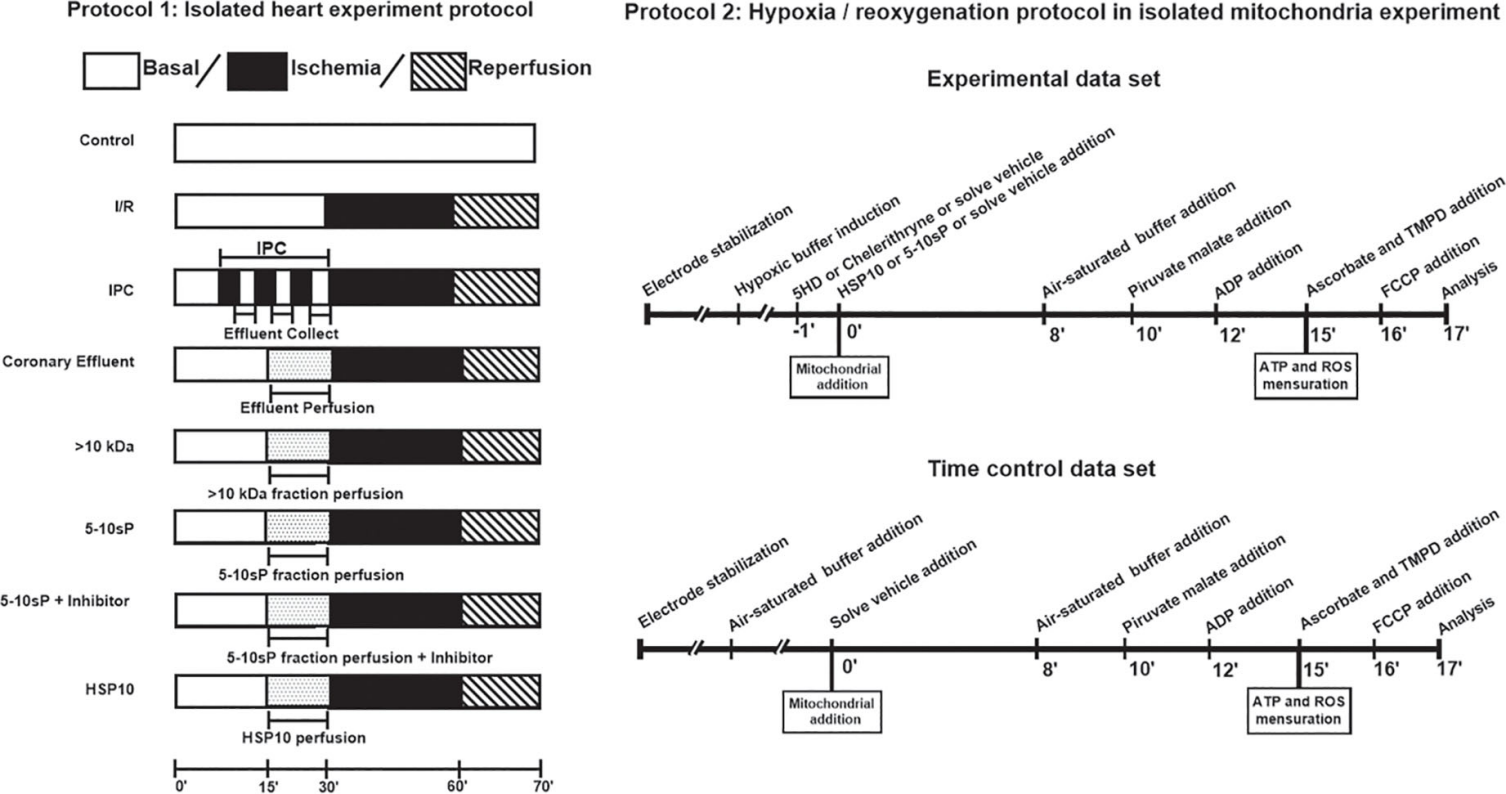
Protocol 1—Isolated heart experiment. IPC hearts were subjected to 3 cycles of 5 min global ischemia/5 min reperfusion. Coronary effluent perfusion (CE) or its fractions were perfused in naíve hearts for 15 min prior to ischemia/reperfusion. HSP10 was administered for 10 min before I/R. 5-hydroxydecanoate (5HD) or chelerythrine (Chel) were administered for 20 min, starting 5 min prior to 5–10 kDa fraction perfusion. Protocol 2—Hypoxia/reoxygenation on isolated mitochondria. The buffer was made hypoxic. Mitochondrial proteins (300 μg) were added to the hypoxic buffer. After 8 min of hypoxia, O_2_-saturated incubation buffer was added to achieve reoxygenation for 2 min. After simulated hypoxia/reoxygenation or the respective normoxic time control (10 min), pyruvate and malate were given as substrates for complex I, mitochondria were stimulated with ADP and respiration was measured over 2 min. Finally, the mitochondria were used to either measure complex IV respiration and maximal uncoupled oxygen uptake, or extramitochondrial ROS concentration, or ATP production.

The I/R experiments were performed on isolated rat hearts as described previously (Maciel et al., 2017; Lindsey et al., 2018). The hearts were excised and mounted on a Langendorff-apparatus and perfused at the constant flow of 10 ml/min with modified Krebs–Henseleit buffer (KHB) (in mmol·L^−1^: NaCl 118.0, NaHCO_3_ 25.0, KCl 4.7, KH_2_PO_4_ 1.2, MgSO_4_ 1.2, CaCl_2_ 1.25, and glucose 11.0) at 37°C and equilibrated with a gas mixture of 95% O_2_ and 5% CO_2_ (pH = 7.4). The perfusate temperature was held constant by a heat exchanger located next to the aortic cannula. A fluid-filled latex balloon was inserted through the left atrium into the left ventricle and connected to a pressure transducer and the PowerLab/400 System (ADInstruments, Australia) for continuous measurement of the left ventricular pressure (LVP). Initial left ventricular end-diastolic pressure was set to 10 mmHg by gradual balloon inflation. Hearts were continuously immersed in 37°C Krebs–Henseleit buffer to avoid hypothermia and allowed to stabilize for 20 min before being subjected to the following protocols (**Figure 1**). Hearts showing LVDP < 80 mmHg or LVDP > 130 mmHg in the baseline period were excluded from this study.

### Protocols

All hearts were submitted to 20 min of the baseline period.

#### Control Experiments (Ctl, n = 5)

Isolated perfused hearts were continuously perfused with saline KHB solution for 70 min.

#### Ischemia/Reperfusion (I/R: n = 5)

Isolated perfused hearts were subjected to 30 min of global zero-flow ischemia and 10 min reperfusion.

#### Ischemic Preconditioning (IPC: n = 5)

Isolated perfused hearts were subjected to 3 cycles of 5 min/5 min global zero-flow ischemia/reperfusion immediately prior to 30 min of ischemia and 10 min of reperfusion. Coronary effluent was collected during the reperfusion episodes of the IPC maneuver and subsequently used (within 2 h).

#### Infusion of Coronary Effluent (CE, n = 5)

Isolated perfused hearts were perfused with coronary effluent from IPC hearts for 15 min before 30 min of global ischemia followed by 10 min of reperfusion.

#### Fractionation and Infusion of >10 kDa-Peptides (> 10 kDa, n = 5)

The coronary effluent was filtered using an ultrafiltration membrane (Amicon model 8200, Millipore) with a cut-off for 10 kDa. The retained fraction was collected and resuspended in 150 ml of Krebs–Henseleit buffer solution. The ultrafiltrated coronary effluent was perfused on the same day of the collection. Isolated perfused hearts were perfused with the >10 kDa peptides fraction for 15 min before 30 min of global ischemia followed by 10 min of reperfusion. The fraction was perfused within 2 h, including ultrafiltration and pH stabilization.

#### Fractionation and Infusion of 5–10 kDa Small Peptides (5–10-sP, n = 5)

The coronary effluent was filtered using ultrafiltration membranes (Amicon model 8200, Millipore) with cut-offs for 5 and 10 kDa. respectively. The IPC coronary effluent was initially ultrafiltrated using the 10 kDa cut-off membrane. The permeated fluid was collected and again ultrafiltrated using the 5 kDa cut-off membrane. The retained was resuspended in 150 ml of Krebs–Henseleit buffer solution. Isolated perfused hearts were perfused with the 5–10 kDa small peptides fraction (5–10-sP) for 15 min before 30 min of global ischemia followed by 10 min of reperfusion. The fraction was perfused within 2 h, including ultrafiltration and pH stabilization.

#### Infusion of 5–10-sP + Hydroxydecanoate (n = 5)

Hydroxydecanoate (5HD) was added to the perfusate at a final concentration of 10 μmol·L^−1^. Isolated perfused hearts were treated with 5HD for 5 min before 5–10-sP infusion and together with 5–10-sP infusion. The protocol was otherwise identical to that for *5*–*10-sP* infusion.

#### Infusion of 5–10-sP + Chelerythrine (n = 5)

Chelerythrine (Chel) was added to the perfusate at a final concentration of 10 μmol·L^−1^. Isolated perfused hearts were treated with Chel for 5 min before 5–10-sP infusion and together with 5–10-sP infusion. The protocol was otherwise identical to that for 5–10-sP infusion.

#### HSP10 (n = 5)

Isolated perfused hearts were perfused with HSP10 1 μmol·L^−1^ (Maciel et al., 2017) for 10 min before 30 min of global ischemia followed by 10 min of reperfusion.

### Mitochondria Isolation and Measurement of Mitochondrial Function

Mitochondria isolation was performed as described previously (Gedik et al., 2017; Bøtker et al., 2018). After 30 min of ischemia and 10 min of reperfusion, the isolated hearts were rapidly removed, placed in ice-cold isolation buffer containing (in mmol·L^−1^): 250 sucrose, 10 HEPES, 1 ethylene glycol tetra-acetic acid (EGTA), pH 7.4 with 0.5% w/v bovine serum albumin (BSA), minced thoroughly using scissors, and then homogenized with a tissue homogenizer (Ultra-Turrax) using two 10 s cycles at a shaft rotation rate of 6,500 rounds per min. This homogenate was further homogenized with proteinase type XXIV (8 IU/mg tissue weight) using a Teflon pestle. The homogenate was centrifuged at 700 g for 10 min at 4°C. The supernatant was collected and centrifuged at 14,000 g for 10 min. The resulting pellet was resuspended in isolation buffer without BSA and centrifuged at 10,000 g for 5 min at 4°C. This procedure was repeated, and the pellet was resuspended in isolation buffer. The protein concentration of the isolated pellet was determined using a protein assay (Lowry method, Biorad, Hercules, CA, USA) by comparison to a BSA standard (Thermo Scientific, Waltham, MA, USA). This methodology was used to isolate the total fraction of mitochondria from cardiac tissue.

### Mitochondrial Oxygen Consumption

Mitochondrial respiration was measured with a Clark-type electrode (Strathkelvin, Glasgow, UK) at 37°C during magnetic stirring in incubation buffer containing in mmol·L^−1^: 125 KCl, 10 MOPS, 2 MgCl_2_, 5 KH_2_PO_4_, 0.2 EGTA with pyruvate (5 mmol·L^−1^), and malate (5 mmol·L^−1^) as substrates for complex I. The oxygen electrode was calibrated using a solubility coefficient of 217 nmol O_2_/ml at 37°C. For the measurement of complex I respiration, the mitochondria (corresponding to a mitochondrial protein amount of 100 µg) were added to 1 ml incubation buffer. After 2 min of incubation, 1 mmol·L^−1^ ADP was added, and ADP-stimulated respiration was measured for 2 min. Afterward, the mitochondria were used to either measure complex IV respiration, and maximal uncoupled oxygen uptake in the respiration chamber or incubation buffer containing the mitochondria was taken from the respiration chamber to measure ATP production or extramitochondrial ROS concentration, respectively. Complex IV respiration was stimulated by adding N,N,N,N′-tetramethyl-p-phenylenediamine (TMPD, 300 μmol·L^−1^) plus ascorbate (3 μmol·L^−1^), which donates electrons to cytochrome oxidase *via* the reduction of cytochrome c. Maximal uncoupled oxygen uptake was measured in the presence of 30 nmol·L^−1^ carbonyl cyanide-p-trifluoromethoxyphenyl-hydrazone (FCCP) (Gedik et al., 2017; Bøtker et al., 2018).

### Mitochondrial Oxygen Consumption Under Simulated Hypoxia/Reoxygenation

The buffer (without pyruvate and malate or succinate) was made hypoxic by the introduction of purified nitrogen until the oxygen concentration was <15 nmol O_2_ ml^−1^. Mitochondria (200 μg) were added to 0.5 ml hypoxic buffer. After 8 min of hypoxia, the O_2_-saturated incubation buffer (0.5 ml) was added to achieve reoxygenation for 2 min. After simulated hypoxia/reoxygenation or the respective normoxic time control (10 min), were added pyruvate (5 mmol·L^−1^) and malate (5 mmol·L^−1^) as substrates for complex I or succinate (5 mmol·L^−1^) as substrates for complex II. Mitochondria were stimulated with ADP and respiration was measured for 2 min. For the measurement of complex II respiration, after hypoxia/reoxygenation, Rotenone (1 µmol·L^−1^) was added. After 2 min of incubation, 1 mmol·L^−1^ ADP was added. Finally, the mitochondria were used to either measure complex IV respiration and maximal uncoupled oxygen uptake, or extramitochondrial ROS concentration, or ATP production. Simulated Hypoxia/Reoxygenation protocols on isolated mitochondria were performed as follows and shown in **Figure 1** (protocol 2).

#### Control (n = 16)

Mitochondria were added to 0.5 ml hypoxic buffer. After 8 min of hypoxia, the O_2_-saturated incubation buffer (0.5 ml) was added to achieve reoxygenation for 2 min. After simulated hypoxia/reoxygenation, the substrates for complex I were given. Finally, the mitochondria were used to either measure complex IV respiration and maximal uncoupled oxygen uptake, or extramitochondrial ROS concentration, or ATP production.

#### Time Control (n = 16)

Mitochondria were added to 0.5 ml oxygenated buffer. After 8 min, O_2_-saturated incubation buffer (0.5 ml) was added for 2 min (time control 10 min), and substrates for complex I were given. Finally, the mitochondria were used to either measure complex IV respiration and maximal uncoupled oxygen uptake, or extramitochondrial ROS concentration, or ATP production.

#### HSP10 (n = 16)

Mitochondrial proteins were added to 0.5 ml hypoxic buffer supplemented with 1 μmol·L^−1^ HSP10. After 8 min of hypoxia, O_2_-saturated incubation buffer (0.5 ml), again supplemented with 1 μmol·L^−1^ HSP10, was added to achieve reoxygenation for 2 min. After simulated hypoxia/reoxygenation substrates for complex I or Rotenone and substrates for complex II were given. Finally, the mitochondria were used to either measure complex IV respiration and maximal uncoupled oxygen uptake, or extramitochondrial ROS concentration, or ATP production.

#### HSP10 + Chelerythrine (n = 8)

Mitochondrial proteins were added to 0.5 ml hypoxic buffer supplemented with 10 μmol·L^−1^ Chelerythrine. After 1 min, was added 1 μmol·L^−1^ HSP10. After 8 min of hypoxia, O_2_-saturated incubation buffer (0.5 ml), again supplemented with 1 μmol·L^−1^ HSP10, was added to achieve reoxygenation for 2 min. After simulated hypoxia/reoxygenation substrates for complex I were given. Finally, the mitochondria were used to either measure complex IV respiration and maximal uncoupled oxygen uptake, or extramitochondrial ROS concentration, or ATP production.

#### HSP10 + 5HD (n = 8)

Mitochondrial proteins were added to 0.5 ml hypoxic buffer supplemented with 10 μmol·L^−1^ 5HD. After 1 min, was added 1 μmol·L^−1^ HSP10. After 8 min of hypoxia, O_2_-saturated incubation buffer (0.5 ml), again supplemented with 1 μmol·L^−1^ HSP10, was added to achieve re-oxygenation for 2 min. After simulated hypoxia/reoxygenation substrates for complex I were given. Finally, the mitochondria were used to either measure complex IV respiration and maximal uncoupled oxygen uptake, or extramitochondrial ROS concentration, or ATP production.

#### 5–10-sP (n = 8)

Mitochondrial proteins were added to 0.5 ml hypoxic buffer supplemented with 5–10-sP. After 8 min of hypoxia, O_2_-saturated incubation buffer (0.5 ml), again supplemented with 5–10-sP, was added to achieve reoxygenation for 2 min. After simulated hypoxia/reoxygenation substrates for complex I or Rotenone and substrates for complex II were given. Finally, the mitochondria were used to either measure complex IV respiration and maximal uncoupled oxygen uptake, or extramitochondrial ROS concentration, or ATP production.

#### 5–10-sP + Chelerythrine (n = 8)

Mitochondrial proteins were added to 0.5 ml hypoxic buffer supplemented with 10 μmol·L^−1^ Chelerythrine. After 1 min 5–10-sP was added. After 8 min of hypoxia, O_2_-saturated incubation buffer (0.5 ml), again supplemented with 5–10-sP, was added to achieve reoxygenation for 2 min. After simulated hypoxia/reoxygenation substrates for complex I were given. Finally, the mitochondria were used to either measure complex IV respiration and maximal uncoupled oxygen uptake, or extramitochondrial ROS concentration, or ATP production.

#### 5–10-sP + 5HD (n = 8)

Mitochondrial proteins were added to 0.5 ml hypoxic buffer supplemented with 10 μmol·L^−1^ 5HD. After 1 min, 1 μmol·L^−1^ 5–10-sP was added. After 8 min of hypoxia, O_2_-saturated incubation buffer (0.5 ml), again supplemented with 5–10-sP, was added to achieve reoxygenation for 2 min. After simulated hypoxia/reoxygenation substrates for complex I were given. Finally, the mitochondria were used to either measure complex IV respiration and maximal uncoupled oxygen uptake, or extramitochondrial ROS concentration, or ATP production.

#### 5HD or Chelerythrine (n = 8)

Mitochondrial proteins were added to 0.5 ml hypoxic buffer supplemented with 1 μmol·L^−1^ 5HD or 10 μmol·L^−1^ Chelerythrine. After 8 min of hypoxia, O_2_-saturated incubation buffer (0.5 ml), again supplemented with 1 μmol·L^−1^ HSP10, was added to achieve reoxygenation for 2 min. After simulated hypoxia/reoxygenation substrates for complex I were given. Finally, the mitochondria were used to either measure complex IV respiration and maximal uncoupled oxygen uptake, or extramitochondrial ROS concentration, or ATP production.

### Mitochondrial ATP Production

After the measurement of ADP-stimulated respiration, the incubation buffer containing mitochondria was taken from the respiration chamber and immediately supplemented with the ATP assay mix (diluted 1:5). Mitochondrial ATP production after each respiration measurement was determined immediately and compared with ATP standards using a 96-well white plate and a spectrofluorometer (SpectraMax^®^ M3, Molecular Devices, EUA) at 560 nm emission wavelength.

### Mitochondrial Swelling and Transmembrane Potential

The mitochondrial swelling and transmembrane potential were evaluated using a spectrofluorometer (SpectraMax^®^ M3, Molecular Devices, EUA). The integrity of the mitochondrial membrane was assessed by osmotically induced volume changes of the mitochondria and spectrophotometric determination of the apparent absorption of the suspension at 540 nm. A mitochondrial suspension (100 μg/ml) was added to the respiration medium in the absence of respiratory substrates, at 37°C and under constant stirring. The mitochondrial turgor was stimulated with 100 nmol·L^−1^ calcium. The swelling was expressed as a percentage of the absorption of the solution containing mitochondria in the presence of cyclosporin A (0% of mitochondrial turgor), in relation to that absorbed after the addition of FCCP (100% of mitochondrial turgor). For mitochondrial transmembrane potential (Δ*ψ*m) determination the probe TMRM (tetramethylrhodamine methyl ester, 400 nmol·L^−1^) was added in incubation solution containing 100 μg/ml of mitochondria for 1 h. The Δψm was estimated by fluorescence emitted by TMRM under 580 nm excitation. The Δψm was expressed as the percentage of fluorescence emitted by TMRM labeled mitochondria in the presence of cyclosporin A (0% of mitochondrial despolarization), relative to that emitted after the addition of FCCP to fully depolarize the mitochondria (100% of mitochondrial despolarization).

### Extramitochondrial ROS Concentration

The Amplex Red Hydrogen Peroxide Assay Kit (Life Technologies, Carlsbad, CA, USA) was used to determine extramitochondrial ROS concentration. Amplex Red reacts at 1:1 stoichiometry with peroxides under catalysis by horseradish peroxidase (HRP) and produces highly fluorescent resorufin. The incubation buffer containing mitochondria was removed from the respiration chamber and immediately supplemented with 50 µmol·L^−1^Amplex UltraRed and 2 U/ml HRP. The supernatant was collected after 120 min incubation in the dark. Extramitochondrial ROS concentration was determined and compared with H_2_O_2_ standards using a 96-well black plate and a spectrofluorometer (SpectraMax^®^ M3, Molecular Devices, EUA) at 540 nm emission and 580 nm extinction wavelengths.

### Statistics

Data are presented as the mean ± standard error of the mean (SEM). Data were analyzed by Prism 6.0 software (GraphPad, San Diego, California, USA) using two-way ANOVA for repeated measures (Left ventricular developed pressure and coronary flow) and one-way ANOVA (Mitochondrial respiration, ATP production, mitochondrial swelling, mitochondrial transmembrane potential, and extramitochondrial ROS concentration). When a significant difference was detected, one-way ANOVA was followed by Bonferroni *post hoc* tests. P < 0.05 was considered statistically significant.

## Results

### Left Ventricular Developed Pressure in Isolated Perfused Rat Hearts

The baseline left ventricular developed pressure was not different among the groups. Control hearts did not show LVDP oscillations during the 40-min normal perfusion. During the preischemic preconditioning, the LVDP values were similar among the groups. The preconditioned groups IPC, coronary effluent, 5–10-sP, and HSP10 presented a greater post-ischemic recovery of LVDP than I/R, > 10 kDa, 5–10-sP + 5HD and 5–10-sP + Chel (**Table 1**).

**Table 1:** Left ventricular developed pressure of isolated perfused rat hearts.

Time	Groups								
	Control (n = 5)	I/R (n = 5)	IPC (n = 5)	Coronary effluent (n = 5)	>10 kDa (n = 5)	5–10-sP (n = 5)	5–10-sP+5HD (n = 5)	5–10-sP+Chel (n = 5)	HSP10 (n = 5)
Baseline 30 min	105.1 ± 13.3	101.5 ± 12.7	104.7 ± 13.8	99.8 ± 7.9	105.3 ± 11. 3	103.9 ± 15.8	104.5 ± 11.4	114.4 ± 15.3	105.4 ± 13.4
Normal flow 40 min	104.0 ± 13.5								
Normal flow 50 min	108.3 ± 14.6								
Normal flow 70 min	103.5 ± 12.4								
After preconditioning			97.6 ± 12.9	107.6 ± 10.7	95. 3 ± 15.9	97.5 ± 9.7	87.4 ± 9.6	106.6 ± 7.8	98.3 ± 9.8
Ischemia 5 min		0.8 ± 0.5	1.0 ± 0.3	1.2 ± 0.0	0.0 ± 0.0	0.0 ± 0.0	0.6 ± 0.0	0.0 ± 0.0	0.0 ± 0.0
Ischemia 30 min		0.0 ± 0.0	0.0 ± 0.0	0.0 ± 0.0	1.1 ± 0.90	0.0 ± 0.0	0.0 ± 0.0	0.0 ± 0.0	0.0 ± 0.0
Reperfusion 10 min		14.6 ± 6.7	51.9 ± 19.6*	46.7 ± 7.6*	19.6 ± 11.8^$^	49.8 ± 8.6*	8.3 ± 7.2^&^	11.3 ± 9.6^&^	47.2 ± 10.3*

Developed left ventricular pressure (LVDP) was calculated as the difference between the systolic and the end diastolic pressure. LVDP values (mmHg) were measured at different time points: at end of stabilization period (baseline), at 40, 50 and 70 min of normal flow in Control hearts, after preconditioning (IPC, perfusion of coronary effluent, 5–10-sP, HSP10, 5HD or chelerythrine), at 5 and 30 min of ischemia, and at 10 min of reperfusion. Mean ± SEM. *P < 0.05 vs I/R, ^$^P < 0.05 vs >10 kDa, ^&^P < 0.05 vs 5–10 kDa.

### Mitochondrial Respiration

**Figure 2:**
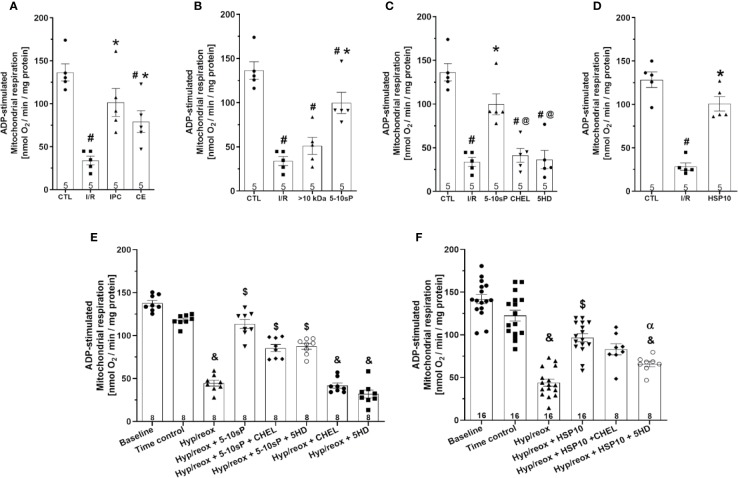
Adenosine diphosphate (ADP)-stimulated complex I respiration of isolated mitochondria from rat hearts. **(A**–**D)** Isolated mitochondria from hearts submitted to I/R. Control (CTL), ischemia/reperfusion (I/R), ischemic preconditioning (IPC), preconditioned coronary effluent transfer (CE), coronary effluent fraction 5–10 kDa (5–10-sP) transfer, coronary effluent fraction >10 kDa (> 10 kDa) transfer, 5–10-sP plus PKC inhibitor chelerythrine or mitochondrial K_ATP_ channel blocker 5HD. **(E**, **F)** Isolated mitochondria from hearts submitted to I/R under 10 kDa-mitochondrial heat shock protein (HSP10) perfusion. Mitochondria respiration submitted to hypoxia/reoxygenation and **(F)** HSP10 or **(E)** 5–10-sP with or without chelerythrine or 5HD incubation. Number in each column is *n* of hearts. ^#^P < 0.05 *vs.* CTL, *P < 0.05 *vs.* I/R, ^@^P < 0.05 *vs.* 5–10-sP, ^&^P < 0.05 *vs.* Time control, ^$^P < 0.05 *vs.* hypoxia/reoxygenation. **^α^**P < 0.05 *vs.* hypoxia/reoxygenation + HSP10.

**Figure 3:**
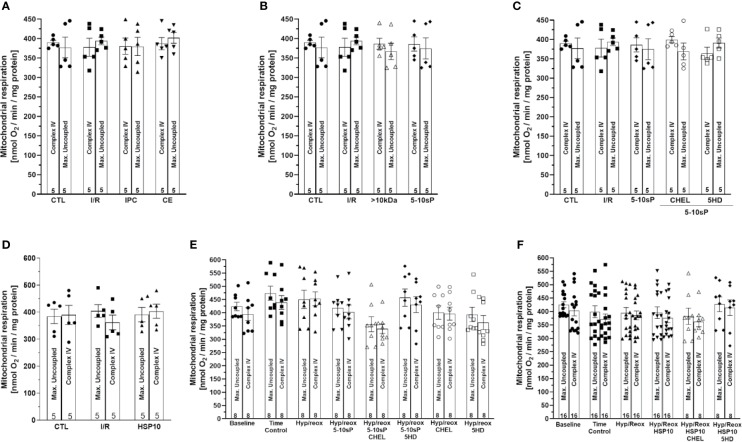
Complex IV respiration with TMPD and ascorbate and maximal uncoupled oxygen uptake with FCCP of isolated mitochondria from rat hearts. **(A**–**D)** Isolated mitochondria from hearts submitted to I/R. Control (CTL), ischemia/reperfusion (I/R), ischemic preconditioning (IPC), preconditioned coronary effluent transfer (CE), coronary effluent fraction 5–10 kDa (5–10-sP) transfer, coronary effluent fraction >10 kDa (> 10 kDa) transfer, 5–10-sP plus PKC inhibitor chelerythrine or mitochondrial KATP channel blocker 5HD. **(E**, **F)** Isolated mitochondria from hearts submitted to I/R under 10 kDa-mitochondrial heat shock protein (HSP10) perfusion. Mitochondria respiration submitted to hypoxia/reoxygenation and **(F)** HSP10 or **(E)** 5–10-sp with or without chelerythrine or 5HD incubation. Number in each column is n of hearts.

ADP-stimulated complex I respiration was reduced after I/R compared to Control (**Figure 2A**). IPC, coronary effluent, 5–10-sP (**Figures 2A, B**) and HSP10 (**Figure 2D**) improved the ADP-stimulated complex I respiration compared to I/R. However, the perfusion of >10 kDa-peptides (**Figure 2B**) or the 5–10 kDa peptides in the presence of chelerythrine or 5HD did not improve the ADP-stimulated complex I respiration (**Figure 2C**). Isolated mitochondria submitted to hypoxia/reoxygenation showed a reduction in ADP-stimulated complex I respiration compared to baseline and the Time control (**Figures 2E, F**). Incubation of isolated mitochondria with HSP10 (**Figure 2F**) or 5–10-sP (**Figure 2E**) prior to hypoxia/reoxygenation induced improvement on ADP-stimulated complex I respiration over that presented by the hypoxia/reoxygenation group. Chelerythrine did not abolish the effects of HSP10 or 5–10-sP despite having a nonsignificant trend to reduce the maintenance of the respiration generated by HSP10 or 5–10-sP. 5HD reduced the respiration maintenance induced by HSP10 but showed a nonsignificant trend to reduce in the 5–10-sP treatment (**Figures 2E, F**). Mitochondrial complex IV respiration and maximal oxygen uptake of uncoupled mitochondria were not different between groups, reflecting an equal loading of viable mitochondria in all experiments (**Figures 3A–F**).

**Figure 4:**
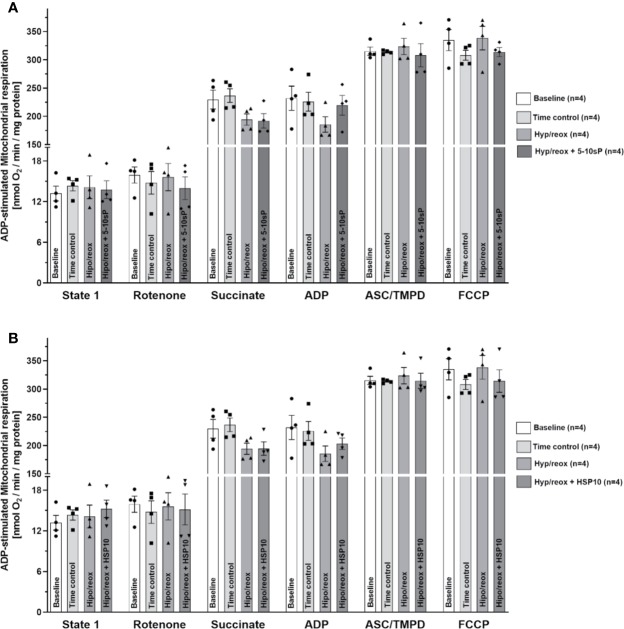
Succinate and adenosine diphosphate (ADP)-stimulated complex II respiration of isolated mitochondria from rat hearts. **(A)** Hypoxia/reoxygenation plus 5–10-sP and **(B)** hypoxia/reoxygenation plus HSP10. State 1—without substrates. Rotenone—Rotenone 1 µmol·L^−1^ was added to the respiration buffer after 1 min. Succinate—Succinate 5 mmol·L^−1^ was added to the respiration buffer after 2 min. ADP—ADP 1 mmol·L^−1^ was added to the respiration buffer after 2 min. TMPD plus ascorbate—TMPD 300 μmol·L^−1^ plus ascorbate 3 μmol·L^−1^ were added to the respiration buffer after 3 min. FCCP—FCCP 30 nmol·L^−1^ was added to the respiration buffer after 1 min. Groups: Baseline, Time control, Hypoxia/reoxygenation (Hyp/reox), Hypoxia/reoxygenation plus HSP10 (Hyp/reox+HSP10), Hypoxia/reoxygenation plus 5–10-sP (Hyp/reox + 5–10-sP).

**Figure 5:**
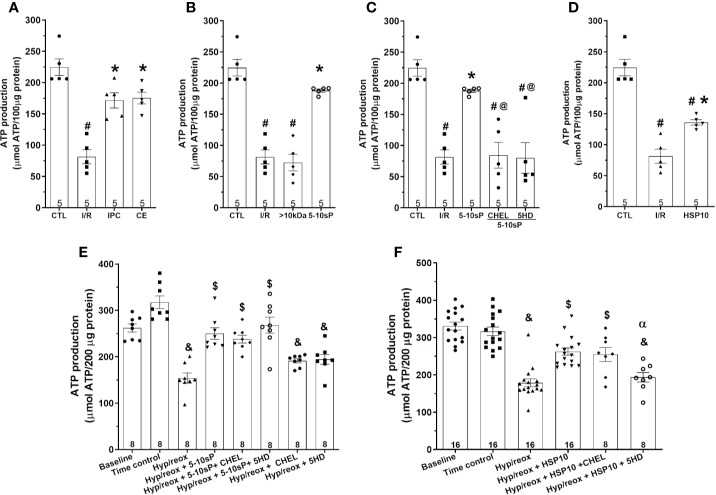
Adenosine triphosphate (ATP) production of isolated mitochondria from rat hearts. **(A**–**D)** Isolated mitochondria from hearts submitted to I/R. Control (CTL), ischemia/reperfusion (I/R), ischemic preconditioning (IPC), preconditioned coronary effluent transfer (CE), coronary effluent fraction 5–10 kDa (5–10-sP) transfer, coronary effluent fraction >10 kDa (> 10 kDa) transfer, 5–10-sP plus PKC inhibitor chelerythrine or mitochondrial K_ATP_ channel blocker 5HD. **(E**, **F)** Isolated mitochondria from hearts submitted to I/R under 10 kDa-mitochondrial heat shock protein (HSP10) perfusion. Mitochondria respiration submitted to hypoxia/reoxygenation and **(F)** HSP10 or **(E)** 5–10-sP with or without chelerythrine or 5HD incubation. Number in each column is *n* of hearts. ^#^P < 0.05 *vs.* CTL, *P < 0.05 *vs.* I/R, ^@^P < 0.05 *vs.* 5–10 kDa, ^&^P < 0.05 *vs.* Time control, ^$^P < 0.05 *vs.* hypoxia/reoxygenation,**^α^**P < 0.05 *vs.* hypoxia/reoxygenation + HSP10.

The complex II respiration did not show a difference between the baseline and the Time control (**Figures 4A, B**). The hypoxia/reoxygenation showed a trend to reduce the state 2-respiration (succinate stimulation) and state 3-respiration (ADP stimulation), however without statistical significance (**Figure 4**). HSP10 (**Figure 4B**) or 5–10-sP (**Figure 4A**) groups did not show effects in complex II. Complex IV respiration and maximal oxygen uptake of uncoupled mitochondria were not different between groups, reflecting an equal loading of viable mitochondria in all experiments (**Figures 4A, B**).

### Mitochondrial ATP Production

The mitochondrial ATP production of the I/R group was diminished compared to the Control group (**Figure 5A**). The IPC, coronary effluent and 5–10-sP groups presented increased ATP production compared to I/R groups (**Figures 5A, B**). Mitochondria from the hearts preconditioned by HSP10 also had an increased ATP production (**Figure 5D**). The >10 kDa peptides did not increase ATP production after I/R (**Figure 5B**). Both chelerythrine and 5HD abrogated the effect of 5–10-sP on ATP production showing values similar to I/R Control (**Figure 5C**). Isolated mitochondria submitted to hypoxia/reoxygenation showed a reduction in ATP production compared to baseline and Time control (**Figures 5E, F**). The mitochondrial ATP production was higher when the isolated mitochondria were incubated with HSP10 or 5–10-sP (**Figures 5E, F**). Chelerythrine did not abolish the effects of HSP10 or 5–10-sP on ATP production. 5HD treatment showed a reduction in the ATP production induced by HSP10 (**Figure 5F**); however, 5HD did not show effect on ATP production induced by 5–10-sP (**Figure 5E**).

### Mitochondrial ROS Production

**Figure 6:**
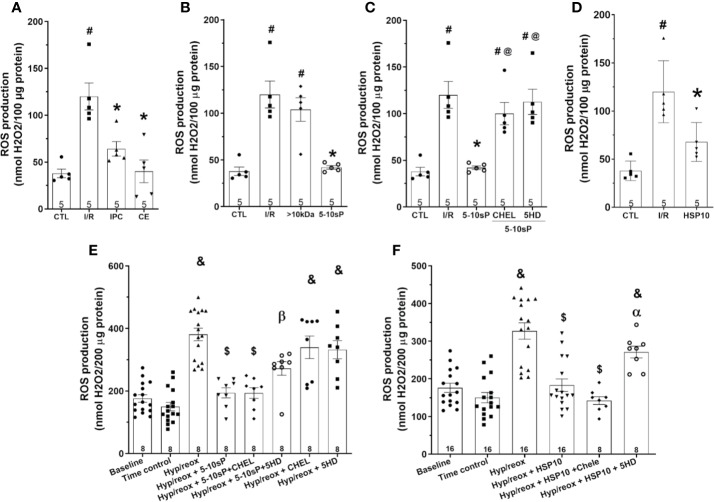
Reactive oxygen species (ROS) production of isolated mitochondria from rat hearts. **(A**–**D)** Isolated mitochondria from hearts submitted to I/R. Control (CTL), ischemia/reperfusion (I/R), ischemic preconditioning (IPC), preconditioned coronary effluent transfer (CE), coronary effluent fraction 5–10 kDa (5–10-sP) transfer, coronary effluent fraction >10 kDa (> 10 kDa) transfer, 5–10-sP plus PKC inhibitor chelerythrine or mitochondrial K_ATP_ channel blocker 5HD. **(E, F)** Isolated mitochondria from hearts submitted to I/R under 10 kDa-mitochondrial heat shock protein (HSP10) perfusion. Mitochondria respiration submitted to hypoxia/reoxygenation and **(F)** HSP10 or **(E)** 5–10-sP with or without chelerythrine or 5HD incubation. Number in each column is *n* of hearts. ^#^P < 0.05 *vs.* CTL, *P < 0.05 *vs.* I/R, ^@^P < 0.05 *vs.* 5–0 kDa, ^&^P < 0.05 *vs.* Time control, ^$^P < 0.05 *vs.* hypoxia/reoxygenation,**^α^**P < 0.05 *vs.* hypoxia/reoxygenation + HSP10, **^β^**P < 0.05 *vs.* hypoxia/reoxygenation + 5–10-sP.

Mitochondrial ROS production after I/R was higher compared to Control (**Figure 6A**). The ROS production in hearts preconditioned with IPC, coronary effluent or 5–10-sP was reduced when compared with that in I/R (**Figures 6A, B**). The HSP10 reduced the mitochondrial ROS production compared to I/R (**Figure 6D**). The >10 kDa peptides did not reduce ROS production after I/R (**Figure 6B**). The 5–10-sP-induced reduction of ROS production was abrogated by chelerythrine or 5HD treatment (**Figure 6C**). The ROS formation on isolated mitochondria was higher on induced hypoxia/reoxygenation than Time control. Incubation of isolated mitochondria with HSP10 reduced ROS formation over that with only hypoxia/reoxygenation (**Figures 6E, F**). Chelerythrine did not abolish the reduction in the ROS generation induced by of HSP10 or 5–10-sP (**Figures 6E, F**). However, 5HD showed a significant increase in the ROS generation of mitochondria incubated with HSP10 under hypoxia/reoxygenation (**Figure 6F**). Furthermore, 5HD increased the ROS generation in the mitochondria incubated with 5–10-sP under hypoxia/reoxygenation (**Figure 6E**).

### Mitochondrial Δψm

**Figure 7:**
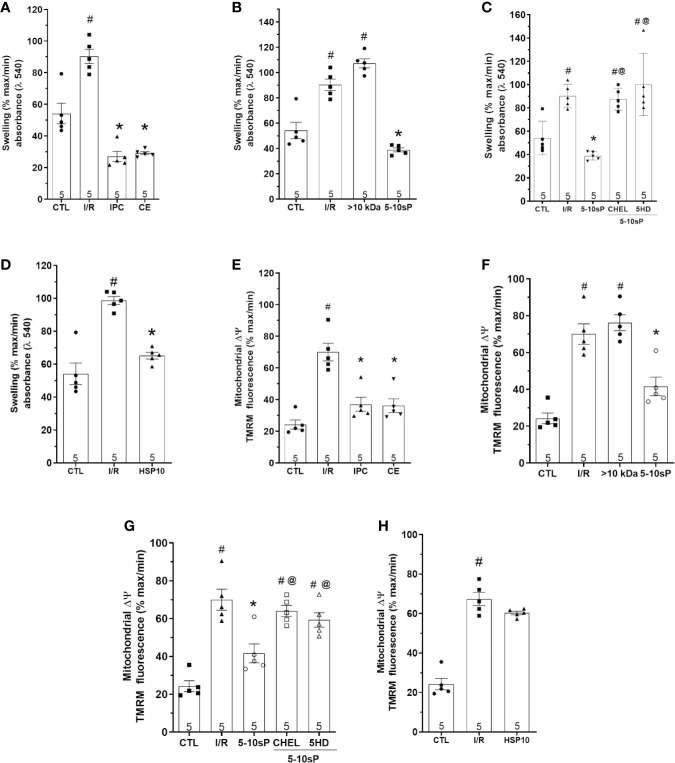
Mitochondrial swelling and mitochondrial transmembrane potential (Δψm) of isolated mitochondria from rat hearts. **(A**–**D)** Mitochondrial swelling and **(E**–**H)** mitochondrial Δψm in isolated mitochondria from hearts submitted to I/R. Control (CTL), ischemia/reperfusion (I/R), ischemic preconditioning (IPC), preconditioned coronary effluent (CE) transfer, coronary effluent fraction 5–10 kDa (5–10-sP) transfer, coronary effluent fraction >10 kDa (> 10 kDa) transfer, 5–10-sP plus PKC inhibitor chelerythrine or mitochondrial K_ATP_ channel blocker 5HD. **(D)** Mitochondrial swelling and **(H)** mitochondrial Δψm in isolated mitochondrial from hearts submitted to I/R, under 10 kDa-mitochondrial heat shock protein (HSP10) perfusion. Number in each column is *n* of hearts. ^#^P < 0.05 *vs.* CTL, *P < 0.05 *vs.* I/R, ^@^P < 0.05 *vs.* 5–10 kDa.

The mitochondrial Δψm was increased after I/R compared to Control (**Figure 7A**). Mitochondrial Δψm depolarization was prevented by IPC and coronary effluent or 5–10-sP perfusion before I/R (**Figure 7B**). However, mitochondrial Δψm depolarization was not prevented by >10 kDa peptides or 5–10-sP perfusion in the presence of chelerythrine or 5HD (**Figures 7B, C**). Contrasting with other results, HSP10 did not show a significant decrease in mitochondrial Δψm (**Figure 7D**).

### Mitochondrial Swelling

Mitochondrial swelling showed the same pattern as mitochondrial ΔΨ. The mitochondrial swelling was prevented by IPC, coronary effluent and 5–10-sP when compared with I/R (**Figures 7E, F**). However, mitochondrial swelling increase after I/R was not prevented by >10 kDa peptides or 5–10-sP perfusion in the presence of chelerythrine or 5HD (**Figures 7F, G**). Different from Δψm, HSP10 reduced mitochondrial swelling over that with I/R (**Figure 7H**).

## Discussion

To the best of our knowledge, this is the first study to describe direct actions of HSP10 on mitochondrial function preservation, since incubation of isolated mitochondria with HSP10 prior to hypoxia/reoxygenation improved the mitochondrial function. Additionally, 5–10-sP from preconditioned coronary effluent and HSP10 maintained mitochondrial function in isolated hearts submitted to I/R and in isolated fresh mitochondria submitted to hypoxia/reoxygenation. These data support the hypothesis that mitochondria are target organelles for humoral factors released during IPC in myocardial tissue.

Ischemic preconditioning is the most effective conditioning maneuver for heart protection and prior studies suggested that IPC is associated with improved mitochondrial respiration and infarct size reduction (Halestrap et al., 2007; Wang et al., 2008; Slagsvold et al., 2014; Kleinbongard et al., 2015; Zhang et al., 2018; Chen et al., 2019; Venkatesh et al., 2019; Yan et al., 2019). In the present study, the improvement in mitochondrial function by HSP10 was comparable to that induced by IPC or preconditioning by Coronary effluent or 5–10-sP perfusion. However, our previous study showed lower cardioprotection by exogenous HSP10 than that conferred by 5–10-sP or IPC (Maciel et al., 2017). Indeed, a previous study showed that remote cardioprotection by IPC effluent transfer preserved the mitochondrial function by a mechanism involving the adenosine receptor (Leung et al., 2014). However, despite adenosine having mimicked the infarct area reduction produced by IPC remote cardioprotection, its global performance was not equal to IPC protection (Leung et al., 2014). These data suggest that other humoral factors are involved in the cardioprotective signaling triggered by IPC (Maciel et al., 2017). Our previous study showed 5–10-sP protecting the myocardium against I/R injuries. In addition, we resolved the identity of several peptides in this molecular weight range of preconditioned coronary effluent (Maciel et al., 2017). Here, we showed that 5–10-sP could actuate directly on mitochondria. However, we do not know the contribution of each peptidic component present in the 5–10-sP in mitochondrial maintenance under hypoxia/reoxygenation (Maciel et al., 2017). Regarding the identification of cardioprotective humoral factors, some of these cardioprotective factors have been identified on IPC and RIPC (Davidson et al., 2013; Kalakech et al., 2014; Heusch, 2015b; Maciel et al., 2017). However, the inhibition of any single component under specific conditions can block the resulting protective effect, and selective activation of that specific component induces protection, a paradox phenomenon in the cardioprotection (Davidson et al., 2013; Jensen et al., 2013; Kalakech et al., 2014; Heusch, 2015b; Maciel et al., 2017). Identifying the humoral factors responsible for RIPC might be useful in developing drugs that confer RIP’s protection in a more efficient and reliable manner (Davidson et al., 2013; Jensen et al., 2013; Kalakech et al., 2014; Heusch, 2015b; Maciel et al., 2017).

The present study showed that exogenous HSP10 or 5–10-sP induces mitochondrial function improvement by direct (in isolated mitochondria) and indirect (in perfused isolated rat hearts) exposure. Although, it remains unclear how HSP10 directly protects the mitochondria against hypoxia/reoxygenation injury, our data suggested that this effect could be mediated by K_ATP_ channels activation. HSP10 has been reported to modulate intracellular cardioprotective signaling such as BCL-2 (Shan, 2003). Furthermore, protection by HSP10 has also been associated with protein folding or ubiquitination (Shan, 2003; Shan et al., 2003) and mitochondrial electron transport chain complexes preservation (Shan, 2003; Shan et al., 2003). Mitochondrial proteins and DNA are vulnerable because of the proximity to the mitochondrial respiratory chain, the main source of ROS production (Lin et al., 2001; Shan, 2003; Shan et al., 2003; Jia et al., 2011). Furthermore, mitochondrial DNA repair capacity is less efficient than in the nucleus (Lin et al., 2001; Shan, 2003; Shan et al., 2003; Jia et al., 2011). This is enhanced during I/R events due to higher ROS production (Heusch and Gersh, 2016; Gedik et al., 2017) and direct and indirect (through DNA) damages to proteins may cause many folding defects. Chaperones such as HSP10 act in protein refolding, translocation and degradation. Therefore, HSP10 is critical in the maintenance of mitochondrial homeostasis under normal and stress conditions (Lin et al., 2001; Shan, 2003; Shan et al., 2003; Jia et al., 2011). A previous study in a murine model showed that the cardioprotection induced by RIPC was related with S-nitrosylation of mitochondrial complex I, reducing its activity and finally reducing myocardial ROS production (Rassaf et al., 2014). However, in the present study, the reduction in ROS production is explained by mitochondrial preservation induced by 5–10-sP and HSP10. When ADP is added on preserved mitochondria, the rate of electron flow strongly increases (Barja, 2007), consequently the electrons flow quickly through the respiratory chain reducing O_2_ to water at cytochrome oxidase and decreasing the degree of electronic reduction of the chain, and thus of its ROS generators (Barja, 2007).

The increased ATP production is an expected consequence of increased ADP-stimulated respiration (Barja, 2007; Doenst et al., 2013). In accordance, increased ATP production was paralleled by the increased respiration induced by 5–10-sP, HSP10-infusion, and 5–10-sP or HSP10-incubated on isolated mitochondria. In addition to improved mitochondrial respiration, 5–10-sP decreased mitochondrial ΔΨ depolarization and swelling. However, HSP10 prevented mitochondrial swelling but did not prevent (statistically) mitochondrial ΔΨ depolarization. Our data suggested possible participation of K_ATP_ channels in the mechanisms by which the HSP10 can protect the mitochondria against injuries by hypoxia and reoxygenation. The activation of these channels creates an influx of potassium (positive charges) into the mitochondrial matrix (Szabò et al., 2012), causing a depolarization of the inner mitochondrial membrane (Szabò et al., 2012). Thus, these results seem to suggest a mechanism by which HSP10 was unable to reduce the mitochondrial membrane potential. This effect favors a reduction in the electric potential difference reducing the protons driving force, thereby preserving the phosphorylating complex during oxygen scarcity. Mitochondrial swelling and ΔΨ depolarization, as a consequence of mPTP opening, increased at early reperfusion (Cohen et al., 2008; Halestrap and Pasdois, 2009; Skyschally et al., 2010). The mPTP opening induces cell death and its inhibition is determinant for cell survival on reperfusion (Cohen et al., 2008; Halestrap and Pasdois, 2009; Skyschally et al., 2010). Previous studies have shown that the inhibition of the mPTP reduces reperfusion injury in porcine models (Skyschally et al., 2010) and also in patients with acute myocardial infarction (Piot et al., 2008). Differently from our study, humoral factor transfer from rabbit plasma submitted to RIPC to isolated neonatal rabbit hearts did not affect the mPTP opening after 30 min reperfusion (Budas et al., 2010). Since mitochondrial swelling is a consequence of mPTP opening (Cohen et al., 2008; Halestrap and Pasdois, 2009; Skyschally et al., 2010) it should not be reversible at late reperfusion (Heusch et al., 2010) explaining these contrasting results.

Our previous study showed that cardioprotection elicited by 5–10-sP was mediated by PKC (Maciel et al., 2017). Here, we investigated whether 5–10-sP activates a PKC-dependent intracellular pathway that converges to the mitochondria, improving its function. Mitochondria from naïve hearts perfused with 5–10-sP in the presence of a PKC inhibitor showed an abrogation of the mitochondrial function improvement. However, PKC inhibitor was not able to abolish the protection induced by HSP10 or 5–10-sP in isolated fresh mitochondria submitted to hypoxia/reoxygenation. This result could be explained by the direct action of these peptides on mitochondrial structures and functions, without depending on a second intracellular pathway. However, it is canonical for cardioprotection that Epsilon-isoform PKC (PKCϵ) acts in IPC mediated protection in rat and murine models (Gray et al., 1997; Ohnuma et al., 2002; Ping et al., 2002; Costa and Garlid, 2008). Furthermore, its translocation to the mitochondria could be dependent on the heat shock proteins (Gray et al., 1997; Ohnuma et al., 2002; Ping et al., 2002; Costa and Garlid, 2008). Once in the mitochondria, PKCϵ activates mitochondrial K_ATP_ channels leading to a small amount of ROS formation, whereas excess formation of ROS contributes to irreversible injury and small amounts of ROS contribute to protection (Heusch, 2015b) through PKCϵ activation, in a feedback positive loop, or by oxidation of others cytosolic kinases (Gray et al., 1997; Ohnuma et al., 2002; Pain et al., 2000; Ping et al., 2002; Heinzel et al., 2005; Costa and Garlid, 2008; Heusch, 2015b). Here, we observe that the blocker for K_ATP_ (5HD) generated an increase in the production of mitochondrial ROS, however, this increase seems to be unrelated to ROS formation in protective amounts, but in an excessive manner that contributes to irreversible injury (Gray et al., 1997; Pain et al., 2000; Ping et al., 2002; Ohnuma et al., 2002; Heinzel et al., 2005; Costa and Garlid, 2008). Indeed, the mitochondrial K_ATP_ blocker reduced the improvement in the mitochondrial function from hearts treated with 5–10-sP. Thus, PKC and mitochondrial K_ATP_ could be involved in mitochondrial function preservation by 5–10-sP. However, it remains unclear whether HSP10 interacts with PKC in the protective effects elicited by 5–10-sP in isolated hearts under I/R (Ohnuma et al., 2002; Costa and Garlid, 2008).Previous studies have shown the participation of the RISK (ERK/AKT) and SAFE (STAT3) pathways in the protection by the transfer of conditioned plasma (Skyschally et al., 2015; Skyschally et al., 2018). Indeed, the activation of these pathways is related to the blockade of the mPTP structuration and protection of the oxidative chain during I/R (Heusch, 2015b; Skyschally et al., 2015; Skyschally et al., 2018). This divergence with our results in the activated pathways could be because here we are using preconditioned coronary effluent and previous studies have used conditioned plasma. Therefore, the humoral factors released by local ischemic preconditioning could be different from those released by remote preconditioning, as well as the activated transduction pathways (Lieder et al., 2018). The released humoral factors seem to be related to the type of conditioning maneuver and the region of the organism subjected to conditioning maneuver (Dickson et al., 1999c; Maciel et al., 2017; Lieder et al., 2018). The neuro-humoral contribution seems to be essential for protection by the RIPC, as well the response through neuro-visceral innervation, *eg*. spleen innervation (Lieder et al., 2018). However, the decisive role of neural/neuro-visceral loop responses does not exclude the cardioprotective contribution of local humoral factors, with no external influence on cardiac tissue (Dickson et al., 1999c; Maciel et al., 2017; Lieder et al., 2018).

### Study Limitation

The lack of data using the PKC inhibitor (chelerythrine) and the blocker for K_ATP_ (5HD) in an isolated heart model treated with HSP10 represents a limitation in clarifying the pathways involved in mitochondrial protection conferred by HSP10. In fact, these experiments were not performed due to a large amount of HSP10 required for the execution of each experiment in an isolated heart model, since the synthesis, purification, and quantification of this amount of protein would take several months. The authors recognize a statistical limitation due to the repetition of some groups in different graphs regarding the assessment of mitochondrial function in hearts undergoing I/R.

In conclusion, our findings provide evidence that the improvement of mitochondrial function by preconditioned coronary effluent transfer is mediated by 5–10-sP humoral factors. This study also supports the mitochondria as an intracellular target of protection by 5–10-sP, and this protection is dependent on the PKC pathway and mitochondrial K_ATP_ channel activation. Finally, this study shows the direct and indirect actions of HS P10 on mitochondrial function maintenance against I/R or hypoxia/reoxygenation injuries.

## Data Availability Statement

The raw data supporting the conclusions of this article will be made available by the authors, without undue reservation.

## Ethics Statement

The animal study was reviewed and approved by the Comissão de Ética no Uso de Animais (CEUA) in scientific experimentation of the Centro de Ciências da Saúde of the Federal University of Rio de Janeiro (100/16) and followed the Guide for the Care and Use of Laboratory Animals published by the US National Institutes of Health (8th edition, 2011).

## Author Contributions

LM, AC, and JN: conception and design, acquisition of data, analysis and interpretation of data, drafting or revising the article, contributed unpublished essential data or reagents. DO and GM: acquisition of data, analysis, and interpretation of data. JN is the principal investigator. All authors discussed the results and commented on the manuscript.

## Funding

This work was supported by the Conselho Nacional de Desenvolvimento Científico e Tecnológico (CNPq) (grant 483639/2013-3 and grant INCT regenera 465656/2014-05) and the Fundação Carlos Chagas Filho de Amparo a Pesquisa do Estado do Rio de Janeiro (FAPERJ) (grant E-26/112.085/2012).

## Conflict of Interest

The authors declare that the research was conducted in the absence of any commercial or financial relationships that could be construed as a potential conflict of interest.

## Abbreviations

AKT, Protein kinase B; FCCP, Carbonyl cyanide-p-trifluoromethoxyphenyl-hydrazone; KHB, Krebs–Henseleit buffer; LVDP, Left Ventricular Developed Pressure; LVEDP, Left Ventricular End-Diastolic Pressure; MPTP, Mitochondrial permeability transition pore; PKC, Protein kinase C; ROS, Reactive Oxygen Species; TMPD, N,N,N,N`-tetramethyl-p-phenylenediamine; Δψ: Mitochondrial transmembrane potential; IPC, ischemic preconditioning; I/R, ischemia/reperfusion; RIPC, remote ischemic preconditioning; 5–10 kDa small peptides (5–10-sP); HSP10, 10 kDa-mitochondrial heat shock protein; LVP, left ventricular pressure; CE, Coronary effluent; Chel, Chelerythrine; 5HD, 5-hydroxydecanoate; PKC***ϵ***, Epsilon-isoform PKC; PTN, Protein.
